# The myths that drive therapeutic inertia in multiple sclerosis: a cost-effectiveness analysis of high-efficacy drugs in Brazil

**DOI:** 10.1055/s-0044-1779036

**Published:** 2024-02-05

**Authors:** Leonardo Zumerkorn Pipek, João Vitor Mahler, Rafaela Farias Vidigal Nascimento, Jefferson Becker, Samira Luísa Apóstolos-Pereira, Tarso Adoni, Guilherme Diogo Silva, Dagoberto Callegaro, on behalf of BCTRIMS (Comitê Brasileiro de Pesquisa e Tratamento em Esclerose Múltipla)

**Affiliations:** 1 Universidade de São Paulo, Hospital das Clínicas, Departamento de Neurologia, São Paulo SP, Brazil.; 2 Universidade de São Paulo, Faculdade de Medicina, São Paulo SP, Brazil.; 3 Faculdade de Medicina do ABC, Centro Universitário, Santo André SP, Brazil.; 4 Pontifícia Universidade Católica do Rio Grande do Sul, Hospital São Lucas, Departamento de Neurologia, Porto Alegre RS, Brazil.

Dear Editor,

We read with great interest the article by Hartmann and colleagues, which argued that the clinical-radiological paradox was a myth in multiple sclerosis (MS). 1 This critical demystification of the clinical-radiological paradox adds a vital element to our growing understanding of the importance of each new lesion in MS. It further underscores the necessity for an aggressive treatment approach to this disease.

Early use of high-efficacy treatments is associated with lower disability scores in the long term. For example, a meta-analysis of large observational studies demonstrated a 30% lower chance of EDSS progression at five years for patients who started high-efficacy disease-modifying drugs as first-line therapy when compared to patients who followed an escalating approach (starting with moderate efficacy drug and upscaling to high-efficacy drug when facing disease activity). 2

However, the early use of high-efficacy treatments is limited by safety and cost concerns. Much like the clinical-radiological paradox, we believe these concerns are other myths that drive therapeutic inertia in MS. A combined analysis of two studies with more than 1,000 patients found no significant difference in the risk of severe adverse events in the group that received early high-efficacy treatment when compared to the escalating strategy. 2 Advances in pharmacovigilance with vaccination, risk stratification for JC virus infection, and infection prophylaxis may explain this finding.

Moreover, the reduction of prices, development of generic drugs, and induction therapies have lowered the cost of high-efficacy treatments. A previously published EDSS-based Markov model suggested the cost-effectiveness of first-line treatment with specific high-efficacy drugs such as natalizumab, alemtuzumab, cladribine, fingolimod, and rituximab. 2 However, this model used the price recommended by the NICE-UK health system, and an analysis using Brazilian disease- and treatment-related costs is necessary.

**Figure 1:**
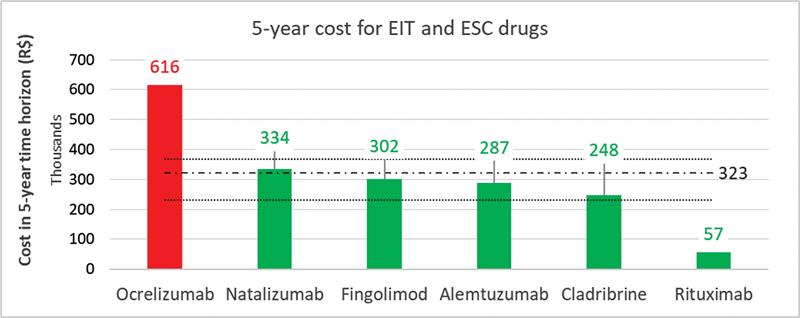
Abbreviations: EIT, Early Intensive Treatment; ESC, Escalating Approach. Notes: red bars represent non-cost-effective drugs, and green bars represent cost-effective drugs; the dashed line indicates the average cost for ESC drugs at R$322.613 (range R$231,349 - R$368,671). 5-year cost analysis of EIT and ESC drugs for the treatment of multiple sclerosis.

We were interested in evaluating whether this finding could be generalized to the context of Brazil. Kobelt et al. published an EDSS-based description of the direct and indirect costs of MS in Brazil. 3 When we applied these values to the same economic model used for the NICE-UK costs, 2 we discovered that early high-efficacy treatment resulted in a decrease of R$2073.00 in non-drug-related costs over 5 years when compared to escalating strategies, owing to the mitigated increase in EDSS. Using the data from the official Brazilian government-suggested prices 4 and the same time horizon, we found the cost of some high-efficacy drugs fell within the same range as the escalating strategy utilizing interferon, glatiramer, teriflunomide, or dimethyl fumarate (ranging from R$231,349 to R$368,671). This was the case for alemtuzumab (R$333.815), cladribine (R$247.968), fingolimod (R$302.038), natalizumab (R$333.815), and rituximab (R$57.244) ( Figure 1 ). Furthermore, rituximab emerged as a cost-effective alternative for treating MS. Despite being an off-label drug for the treatment of MS in Brazil, previous experiences in other countries and randomized controlled trials have shown promising results 5

In conclusion, with the growing understanding that even minor evidence of disease activity can contribute to long-term disability, the need for aggressive treatment is becoming increasingly apparent. It's important to note that aggressive treatment may not present more risks or be more costly than escalating strategies. It is imperative, therefore, to dismantle the myths that perpetuate therapeutic inertia.
